# THE FRAILTY INDEX CONSTRUCTED USING FEATURE SELECTION METHODS IS THE BEST MEASURE OF BIOLOGICAL AGE

**DOI:** 10.1093/geroni/igz038.3261

**Published:** 2019-11-08

**Authors:** Sangkyu Kim, Jessica Fuselier, David Welsh, Katie E Cherry, Leann Myers, S Michal Jazwinski

**Affiliations:** 1 Tulane University Health Sciences Center, New Orleans, Louisiana, United States; 2 Louisiana State University Health Sciences Center, New Orleans, Louisiana, United States; 3 Louisiana State University, Baton Rouge, Louisiana, United States

## Abstract

No single biomarker can reliably represent the complexity of aging. One way to overcome this shortcoming is to aggregate multiple biomarkers into a composite index. The frailty index (FI), which is simply the proportion of accumulated deficits among a set of various health markers, reflects functional abilities and risks of adverse outcomes. Furthermore, the FI accounts for the variation in mortality among individuals of the same chronological age (CA). Thus, the FI is a reliable measure of biological age (BA). Unlike the FI, other popular BA-estimating algorithms use CA directly as a biomarker or indirectly to derive model parameters. However, genetic, pharmaceutical, and intervention studies have shown that aging is delayable or reversible, indicating that CA is not the direct cause of aging. The popular Klemera-Doubal (K-D) method proposes two equations for BA estimation: BE uses CA to derive equation parameters, and BEC directly incorporates CA as an additional biomarker. BA estimates by the K-D method, especially by BEC, have been shown to outperform CA. Using Louisiana Healthy Aging Study (LHAS) data, we constructed an FI from a battery of health items selected using machine learning methods for their ability to predict mortality. We compared the FI with CA and the two K-D BA estimates and found that the FI was the better predictor of mortality, especially among nonagenarians. The results were replicable with the FI calculated from different sets of selected health items using NHANES and HRS datasets. These results demonstrate the FI as the best-performing measure of BA.

